# Trauma and pain sensitization in youth with chronic pain

**DOI:** 10.1097/PR9.0000000000000992

**Published:** 2022-03-16

**Authors:** Joel Janssen, Elias Abou-Assaly, Nivez Rasic, Melanie Noel, Jillian Vinall Miller

**Affiliations:** a Department of Anesthesiology, Perioperative, and Pain Medicine, University of Calgary, Calgary, AB, Canada; b Vi Riddell Children's Pain and Rehabilitation Centre, Alberta Children's Hospital, Calgary, AB, Canada; c Department of Psychology, University of Calgary, Calgary, AB, Canada; d Child Brain & Mental Health Program, Alberta Children's Hospital Research Institute, Calgary, AB, Canada; e Brain & Mental Health, Hotchkiss Brain Institute, Calgary, AB, Canada.

**Keywords:** Chronic pain, Pediatric pain, Posttraumatic stress symptoms, PTSD, Sensitization

## Abstract

**Introduction:**

Chronic pain (pain lasting ≥3 months) co-occurs with internalizing mental health issues, such as posttraumatic stress symptoms (PTSS), at high rates in youth. The mechanisms underlying these relationships remain unclear. Posttraumatic stress symptoms, including re-experiencing (eg, intrusive memories), alterations in cognition and mood, hyperarousal, and avoidance could lead to altered neuronal processing, pain sensitization, and greater reports of pain. However, the relationships between PTSS and pain sensitization in youth with chronic pain are not known.

**Methods:**

Youth (n = 165) aged 10 to 18 years were recruited from outpatient multidisciplinary chronic pain programs. Symptoms of PTSS were assessed using psychometrically sound questionnaires. Youth also underwent a cold-pressor task, the most commonly used experimental pain induction technique. During this task, they reported on their expected pain, actual pain intensity, and pre- and post-state pain catastrophizing. Their pain threshold was recorded. A multivariate general linear model was used to examine the relationships between PTSS, ratings of pain intensity, state pain catastrophizing, and pain threshold, controlling for age, gender, ethnicity, anxiety, and depressive symptoms.

**Results:**

Higher PTSS were associated with greater pain thresholds (*P =* 0.03) and higher pre- and post-state pain catastrophizing (*P* ≤ 0.05).

**Conclusions:**

Individuals with higher PTSS may avoid or dissociate from pain-inducing stimuli, thus leading to higher pain thresholds. However, individuals with higher PTSS also tend to catastrophize prior to and following exposure to pain. Avoidant and pain catastrophizing behaviors may serve to perpetuate chronic pain conditions. Future research is needed to determine how PTSS are related to pain sensitization prior to the development of chronic pain in at-risk youth.

## Abstract 2

Greater posttraumatic stress symptoms were associated with higher experimental pain tolerance. Pain may trigger dissociation in youth with chronic pain and higher posttraumatic stress symptoms.

## 1. Introduction

Pediatric chronic pain is a prevalent condition associated with significant social and economic burden.^19,25,36,37^ It is highly comorbid with internalizing mental health conditions, including posttraumatic stress disorder (PTSD), as well as anxiety and depression.^1,6,34,35^ Youth with chronic pain are at greater risk of receiving a diagnosis of PTSD, anxiety, and depressive disorders into adulthood,^22,33,41,48^ and early exposure to trauma has been associated with an increased risk of developing chronic pain conditions.^39,40^ However, despite sufficient evidence that a relationship between trauma and chronic pain exists, it remains unclear how exposure to trauma leads to the development and maintenance of chronic pain conditions.

Conceptual frameworks have been proposed to explain the co-occurrence of PTSD symptoms (PTSS) and chronic pain, including potentially shared, bidirectional, and mutually maintaining interpersonal and neurobiological factors, as well as individual processes, symptoms, and behaviours.^1,21,46^ At the neurobiological level, trauma may increase the risk for development of chronic pain via changes in shared brain circuitry that become altered following prolonged exposure to trauma symptoms. Recently, we demonstrated that greater PTSS, smaller amygdala (ie, region involved in fear processing) volumes, and altered corticolimbic connectivity were associated with greater average headache frequency in youth with chronic headache.^29^ Indeed, others have also found that as an individual transitions from an acute to chronic pain state, resting brain activity moves from primarily within somatosensory regions to limbic regions, representing a shift from physical to emotional processing.^23,30,42^ Trauma-induced alterations to corticolimbic circuitry may result in altered cognitive–emotional processing, thereby influencing pain sensitivity.

Pain catastrophizing (ie, the tendency to magnify, ruminate, and feel helpless regarding the threat of pain^31^) may lead to increased bodily sensations, which could potentially lead to higher pain intensity and pain sensitivity following exposure to a painful stimulus.^21^ Previously, we have demonstrated that pain catastrophizing mediates the relationship between PTSS and chronic pain intensity in youth with chronic pain.^31^ Therefore, PTSS and pain catastrophizing may work synergistically to exacerbate pain experiences,^31^ which may therein increase pain sensitization and maintain chronic pain conditions.^13,24^

To date, research exploring the influence of trauma on pain sensitization has revealed conflicting results. A recent meta-analysis of the adult literature found that combat-related PTSD was associated with increased pain thresholds, whereas accident-related PTSD was associated with decreased pain thresholds.^43^ However, the relationships between PTSS and pain sensitization in youth with chronic pain remains unknown. In the present study, we examined the relationships between PTSS, state pain catastrophizing, pain intensity, and pain thresholds in youth with chronic pain. We hypothesized that youth with greater PTSS would demonstrate higher state pain catastrophizing, greater expected and experienced pain, and lower pain thresholds as compared with youth with lower PTSS and chronic pain. Understanding the impact of PTSS on pain sensitization may lead to a greater understanding of the mechanisms underlying the development and maintenance of chronic pain conditions in youth.

## 2. Methods

This study was approved by the University of Calgary's Health Research Ethics Board (REB15-3100) and conducted in accordance with the Declaration of Helsinki. A parent of each participant provided informed and written consent. Youth younger than 14 years provided informed and written assent, and youth older than 14 years provided informed and written consent.

### 2.1. Participants

Youth (N = 190) aged 10 to 18 years were recruited from the outpatient, multidisciplinary, chronic pain programs at a tertiary-level children's hospital in Western Canada. Youth were eligible if they identified as having chronic pain (pain lasting ≥3 months), without underlying disease (eg, juvenile arthritis or cancer), and they reported ongoing pain (pain intensity >0/10 in the past month) upon recruitment. Youth were excluded if they were unable to read/speak English, unable to access the Internet, or if they had severe cognitive impairment, developmental disorders, psychotic disorders, attention deficit hyperactivity disorder, and/or the presence of serious chronic health and/or life-threatening conditions (eg, cancer).

### 2.2. Procedure

Contact information of new chronic pain patients, as well as patients who had received care in the chronic pain clinics within the past 2 years, were provided to the research team with permission from the patients by the clinical and/or administrative team. A research assistant subsequently called the family to explain the research study, screen for eligibility, and acquire informed consent. If eligible and interested, a laboratory visit was scheduled, and the parent and youth were sent consent forms via an emailed link to REDCap,^20^ a secure online Web-based application. After clicking “Yes,” on their consent form, youth and one of their parents were sent questionnaires to complete. Parents completed a brief measure of demographics. Youth completed measures of PTSD,^17^ and anxiety and depressive symptoms.^9^ Approximately 1 week later, youth and their parent came to the children's hospital for a laboratory visit. During this visit, youth participated in an experimental pain task (the cold pressor task [CPT]^4^). The CPT is the most commonly used experimental pain induction technique in pediatric pain research.^5^ It measures pain sensitivity variables such as pain intensity and pain tolerance. In accordance with ethical guidelines for administering CPT in children and adolescents, youth were asked to submerge their hand in temperature-controlled cold (10 °C) water for up to 4 minutes.^4,47^ They were instructed that they could take their hand out of the water at any time if it got too painful or uncomfortable to leave it in and therefore had complete control over the task. Prior to the task, their expected pain intensity and state pain catastrophizing score were recorded. During the task, their pain threshold (length of time in the water) was recorded. After the task, their pain intensity score and state pain catastrophizing score were again recorded.

### 2.3. Measures

#### 2.3.1. Demographics

Parents completed a demographic questionnaire, including questions assessing youth age, gender, ethnicity, duration of their pain problem, diagnosis (if applicable), and their family's household annual income.

#### 2.3.2. Posttraumatic stress symptoms

Posttraumatic stress symptoms were assessed using the Child PTSD Symptom Scale (CPSS-V).^17^ The CPSS-V is a 20-item measure that maps on to the Diagnostic and Statistical Manual of Mental Disorders Fifth Edition posttraumatic stress disorder criteria^2^ and assesses PTSS experienced by youth in the past month. Youth were asked to identify the most distressing or traumatic event that bothers them to think about. With that event in mind, they were asked to respond to 20 items assessing PTSS on a 5-point Likert scale, ranging from 0—“not at all” to 4—“6 or more times a week/almost always.” Total symptom severity scores were obtained by summing the 20 items (range: 0–80), with higher scores indicating higher PTSS. A score of 31 or above indicates clinically elevated PTSS. The CPSS-V has demonstrated excellent internal consistency, good test–retest reliability, and good convergent validity.^17^ The Chronbach alpha for this scale was a = 0.95.

#### 2.3.3. Anxiety and depressive symptoms

Youth anxiety and depressive symptoms were assessed using the Revised Child Anxiety and Depression Scale. Youth rank the frequency of their symptoms on a 4-point Likert scale ranging from 0—“never” to 3—“always.” This scale includes 47-items, which form 6 subscales examining (1) separation anxiety disorder, (2) social phobia, (3) generalized anxiety disorder, (4) panic disorder, (5) obsessive compulsive disorder, and (6) low mood (major depressive disorder). The total anxiety score is the sum of the 5 anxiety subscales, and the depression score is the sum of the remaining sixth subscale. A total internalizing score (sum of all 6 subscales) of 70 represents the clinical cutoff.^8^ The clinical threshold maps on to the anxiety disorders interview schedule for Diagnostic and Statistical Manual of Mental Disorders Fourth Edition.^2,8^ The Revised Child Anxiety and Depression Scale has good internal consistency, test–retest reliability, and concurrent and convergent validity.^8,15^ The Chronbach's alpha for this scale was a = 0.97.

#### 2.3.4. Pain catastrophizing and pain intensity

Prior to starting the cold-pressor task, youth rated how much pain they expected to feel (pain intensity) on an 11-point numerical rating scale (NRS) ranging from 0—“no pain” to 10—“worst pain possible.” State pain catastrophizing was measured based on the total score of the following 3 NRS items, as was done previously.^16^ Youth were asked prior to the cold-pressor task: (1) at this moment to what extent do you keep on thinking about the pain you will experience during the cold water task (rumination)?; (2) At this moment to what extent do you think something serious might happen to you because of the pain (magnification)?; (3) At this moment, to what extent do you think you will not be able to stand the cold water task because of the pain (helplessness)? Youth responded to these questions on an 11-point NRS ranging from 0—“not at all” to 10—“a lot”. Following the cold-pressor task, youth rated how much pain they felt (pain intensity) during the CPT, and they were asked the same state pain catastrophizing questions. Previously, we compared state and trait pain catastrophizing and found that child state pain catastrophizing was a better predictor of child pain intensity in response to child acute experimental pain.^16^ Moreover, state pain catastrophizing was a stronger predictor of pain-related outcomes.^16^ Hence, this is why state, rather than trait, pain catastrophizing was used in the present study. The Chronbach alpha for this scale was a = 0.86.

### 2.4. Data analysis

Analyses were conducted using the Statistical Package for the Social Sciences (SPSS) version 26.0.^10^ The data were assessed for the assumptions of normality, including outliers, collinearity of data, independent errors, random normal distribution of errors, homoscedasticity and linearity of data, and non-zero variances. No violations were detected. Analyses were conducted using two-tailed hypothesis testing. Repeated measures ANOVAs were used to explore the differences in pain intensity and state pain catastrophizing ratings before and after CPT. Given that the outcomes are closely related, a multivariate general linear model was performed examining the relationships between PTSS and pain intensity before and after the CPT, pain tolerance, and state pain catastrophizing before and after the CPT, accounting for youth age, gender, and ethnicity variables. Anxiety and depression scores were also included in the multivariate general linear model, as these symptoms can co-occur with PTSS and controlling for them enabled us to isolate the unique predictive value of PTSS.

## 3. Results

### 3.1. Characteristics of the cohort

**Figure 1.**
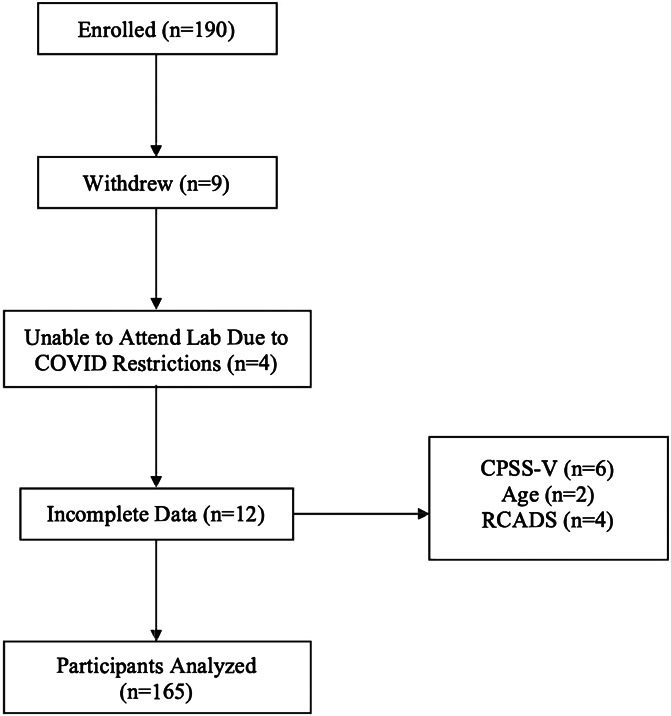
Participant flow chart. CPSS, Child PTSD Symptom Scale; RCADS, Revised Child Anxiety and Depression Scale.

A participant flow chart can be found here (Fig. 1). Overall, 190 participants were enrolled for the study, but 9 withdrew before laboratory testing and 4 were unable to attend laboratory testing due to COVID-19 pandemic restrictions. Data were missing on 12 participants, including 6 scores for PTSS, 3 scores for MCADS, and age demographics for 3 participants. Therefore, a total of 165 participants were included in the analyses. Of the 25 youth not included in the study sample: n = 2 were black (8% compared with 0.6% in the study sample), n = 14 were white (56% compared with 79.4% of the study sample), n = 1 was of mixed ethnicity (4% compared with 12% of study sample), and n = 8 did not want to specify (32% compared with 0.6% of the study sample). There were no significant differences in child age, child gender, or income between participants who were included and participants who were not included. There were also no significant differences in age, gender, ethnicity, or household income between the individuals missing PTSS, anxiety, depression, and/or cold-pressor data. The characteristics of the cohort are described in Table 1. The inciting traumatic events identified by youth are listed in Table 2. Thirty-one youth (18.8%) met the clinical cutoff for PTSS. Of the youth who identified a distressing or traumatic event (n = 126), 110 youth gave either an approximate or specific date for their traumatic experience. Fifty-three youth (48%) identified a distressing or traumatic event that occurred at least 3 months prior to the onset of their chronic pain problem. The commonly reported traumatic event was death (ie, 17% of youth) of either a close family/friend or risk of death to themselves or someone they cared about. Youth with the highest mean PTSS reported physical abuse as the most distressing or traumatic event that bothers them to think about (M = 32, SD = 27).

**Table 1:** Cohort characteristics.

Characteristics	Total (n = 165)
Age, M (SD), y	14.2 (2.3)
Gender (female),n (%)	119.0 (72.1)
Ethnicity (white), n (%)	131.0 (79.4)
Income (>$90,000), n (%)	93.0 (56.4)
Primary pain location(s), n (%)	
Stomach	32.0 (19.4)
Head	120 (72.7)
Muscle and joints	42 (25.5)
Legs	21 (12.7)
Chest	17 (10.3)
Other	41 (24.8)
Self-reported symptoms, M (SD)	
Anxiety symptoms	30.9 (21.0)
Depressive symptoms	9.6 (6.4)
PTSS	16.8 (16.7)
Cold-pressor task, M (SD)	
Pain expectation	3.8 (2.1)
State catastrophizing (before)	4.7 (5.1)
Pain threshold (min)	2.6 (1.6)
Pain intensity	4.2 (2.3)
State catastrophizing (after)	5.0 (5.7)

Missing data: income = 9; pain intensity = 2; state catastrophizing (before) = 2.

Clinical cutoffs: RCADS = 20 (12.1%); CPSS-V = 31 (18.8%).

CPSS-V, Child PTSD Symptom Scale; PTSS, posttraumatic stress symptoms; RCADS, Revised Child Anxiety and Depression Scale.

**Table 2:** Traumatic events identified by youth.

Traumatic event	N = 165 (n [%])	PTSS (mean [SD])
1. Death	28 (17.0)	22.0 (14.8)
2. Fear/anxiety	16 (9.7)	21.8 (17.4)
3. Physical illness or hospitalization	14 (8.5)	9.0 (7.9)
4. Accident	12 (7.3)	15.3 (17.7)
5. Family-related conflict	10 (6.1)	30.6 (22.8)
6. Pet or animal death, illness, or injury	8 (4.8)	10.8 (10.7)
7. Physical abuse	6 (3.6)	32.0 (27.0)
8. Chronic pain problem	5 (3.0)	20.2 (8.9)
9. Social difficulties	5 (3.0)	23.2 (16.8)
10. Academic difficulties	5 (3.0)	28.8 (13.9)
11. Divorce	4 (2.4)	17.8 (19.4)
12. Sexual abuse	4 (2.4)	28.3 (23.1)
13. Verbal conflict or abuse	2 (1.2)	6.0 (5.7)
14. Natural disaster/storms	2 (1.1)	13.5 (13.4)
15. Mental health problems	2 (1.1)	26.0 (4.2)
16. Gun threat/lockdown	2 (1.1)	13.0 (9.9)
17. Fire	1 (0.6)	20.0 (N/A)
18. None	18 (10.9)	0.0 (0.0)
19. Did not specify	21 (12.7)	11.3 (12.2)

PTSS, posttraumatic stress symptoms.

### 3.2. Pain intensity and state pain catastrophizing before and after the cold-pressor task

Expected pain intensity ratings (M = 3.7, SD = 1.2) prior to the CPT were significantly lower than the actual pain intensity ratings (M = 4.1, SD = 2.3), following the CPT (*F*(1,174) = 6.42, *P* = 0.01). State pain catastrophizing, however, did not significantly differ from pre–CPT (M = 4.6, SD = 5.1) to post–CPT (M = 4.9, SD = 5.6; *F*(1,174) = 0.57, *P* = 0.45).

### 3.3. Posttraumatic stress symptoms as a predictor of pain perception and sensitivity

#### 3.3.1. Posttraumatic stress symptoms and pain expectancy

The multiple general linear regression revealed that after controlling for covariates (ie, age, gender, ethnicity, and anxiety and depressive symptoms), PTSS was not associated with pain intensity expectancy. Rather, female gender (ß = −0.27, *t*(160) = −3.77, *P <* 0.001) explained 17% of the variance in the pain expectancy ratings (Table 3).

**Table 3:** Posttraumatic stress symptoms, expected pain, and pain threshold.

	Expected pain intensity (N = 161)			Pain threshold (N = 161)		
	ß	SE	*P*	ß	SE	*P*
Age	0.01	0.08	0.87	0.32	0.08	<0.001
Gender	−0.27	0.07	<0.001	−0.07	0.08	0.35
Ethnicity	0.08	0.07	0.30	0.08	0.08	0.30
Anxiety symptoms	0.04	0.12	0.77	0.02	0.13	0.87
Depressive symptoms	0.14	0.12	0.25	−0.14	0.13	0.29
PTSS	0.12	0.11	0.29	0.26	0.11	0.03
	R^2^ = 17			R^2^ = 16		

ß, beta; N, number; *P*, probability value; PTSS, posttraumatic stress symptoms; R^2^, coefficient of multiple determination.

#### 3.3.2. Posttraumatic stress symptoms and state pain catastrophizing prior to the cold-pressor task

The multiple general regression revealed that over and above covariates (ie, age, gender, ethnicity and anxiety and depressive symptoms), higher PTSS (ß = 0.36, *t*(160) = 3.28, *P =* 0.001) were associated with greater state pain catastrophizing prior to the CPT. Posttraumatic stress symptoms explained 24% of the variance in pain catastrophizing about the CPT–related pain (Table 4).

**Table 4:** Posttraumatic stress symptoms and state pain catastrophizing prior to and after the cold-pressor task.

	State pain catastrophizing prior to the cold-pressor task (N = 161)			State pain catastrophizing after the cold-pressor task (N = 161)		
	ß	SE	*P*	ß	SE	*P*
Age	−0.06	0.08	0.42	−0.09	0.08	0.31
Gender	−0.14	0.07	0.06	<0.001	0.08	>0.99
Ethnicity	0.02	0.07	0.78	−0.05	0.08	0.50
Anxiety symptoms	0.18	0.12	0.16	0.20	0.13	0.13
Depressive symptoms	−0.02	0.12	0.85	−0.09	0.13	0.51
PTSS	0.36	0.11	0.001	0.23	0.12	0.05
	R^2^ = 24			R^2^ = 11		

ß, beta; N, number; *P*, probability value; PTSS, posttraumatic stress symptoms; R^2^, coefficient of multiple determination.

#### 3.3.3. Posttraumatic stress symptoms and pain threshold

The multiple general linear regression revealed that over and above covariates (ie, gender, ethnicity, and anxiety and depressive symptoms), older youth (ß = 0.32, *t*(160) = 3.95, *P <* 0.001) with higher PTSS (ß = 0.26, *t*(160) = 2.26, *P =* 0.03) had higher pain thresholds; thereby, they were able to submerge their hand for a longer period of time during the CPT. Age and PTSS explained 16% of the variance in pain threshold (Table 3).

#### 3.3.4. Posttraumatic stress symptoms and pain intensity

None of the variables that were included in the multiple general linear regression model (ie, age, gender, ethnicity, anxiety and depressive symptoms and PTSS) were associated with pain intensity ratings following the CPT (*F*(6,160) = 0.87, *P* = 0.52).

#### 3.3.5. Posttraumatic stress symptoms and State Catastrophizing after to the cold-pressor task

The multiple general linear regression revealed that over and above covariates (ie, age, gender, ethnicity, and anxiety and depressive symptoms), higher PTSS (ß = 0.23, *t*(160) = 1.97, *P =* 0.05) were associated with higher state pain catastrophizing after the CPT. Posttraumatic stress symptoms explained 11% of the variance in pain catastrophizing about the CPT–related pain (Table 4).

## 4. Discussion

In youth with chronic pain, we examined the relationships between PTSS and state pain catastrophizing, pain intensity, and pain tolerance. Higher PTSS were associated with greater state pain catastrophizing and greater pain thresholds, as compared with individuals with lower PTSS, after accounting for age, gender, ethnicity, and anxiety and depressive symptoms. Posttraumatic stress symptoms were not associated with ratings of pain intensity. Therefore, although individuals reported similar ratings of cold-pressor pain regardless of their level of PTSS, their response and interpretation of that pain appeared to differ if they had higher vs lower levels of PTSS.

A recent meta-analysis of pain processing and perception in healthy adults revealed no significant differences in pain intensity ratings or pain tolerance in individuals with vs without PTSS.^43^ However, when they stratified their sample by individuals with combat-related trauma and accident-related trauma, they found significantly increased and decreased thresholds for pain, respectively.^43^ Therefore, youth with higher PTSS and chronic pain appear to be more similar to adults that have experienced combat-related trauma in their responses to the CPT. The frontal cerebral cortex is the area of the brain that governs rational thought. It undergoes a critical period of development, during adolescence.^26^ Therefore, given the immaturity of their frontal cortex, youth may be less able to handle the complexity of emotions associated with their trauma and pain symptoms, thereby resulting in responses resembling those of adults exposed to severe trauma.

Posttraumatic stress symptoms are characterized by reexperiencing intrusive distressing recollections, avoidance of trauma-related thoughts, alterations in cognition and mood, and hyperarousal and reactivity.^3^ In the present study, the threat of re-experiencing pain in youth with chronic pain and higher PTSS was associated with increased symptoms of pain catastrophizing (ie, magnification, rumination, and helplessness) in contrast with individuals with lower PTSS. Moreover, youth with chronic pain and higher PTSS also had relatively higher pain thresholds during the CPT. These findings were supported by Defrin et al. who found that although individuals with combat-related PTSD rated noxious stimuli as more intense, they had greater pain thresholds relative to the healthy controls.^11,12^ The higher pain ratings among veterans were related to greater reports of anxiety sensitivity, and their higher pain thresholds were associated with higher levels of dissociation.^12^ Further evidence of a stress-related dissociation was demonstrated in veterans with PTSD whom exhibited a larger increase in pain threshold after a cognitive stress task compared with veterans without PTSD.^45^ Therefore, PTSS appear to drive both behavioral and physiological responses to experimental pain.

Stress-induced analgesia may also underlie the increased pain threshold observed in youth with higher vs lower PTSS. Studies examining neural responses to noxious stimulation in adults with and without PTSD have reported increased prefrontal and hippocampal activity,^14,18,27^ which may incite an endogenous opioid-mediated inhibitory cascade, thereby modifying pain responses.^14,45^ Stress-induced analgesia has been shown to be followed by amygdala deactivation,^18,38^ resulting in subsequent hyperarousal.^28,32^ Evolutionarily, these responses to stress and pain are adaptive.^7^ Upon facing an immediate threat, attending to an injury would not be conducive to survival. However, once that threat has been alleviated, sensitization garnering attention would be beneficial for signalling that an injury has occurred and that area of the body should be protected to avoid further aggravation of the injury. Outside of this context, however, stressed-induced analgesia may serve to perpetuate chronic pain symptoms in youth. Indeed, attention to threat, a known characteristic of PTSS, may increase the likelihood of engaging in catastrophic thinking about pain.^31^ Pain catastrophizing has shown to mediate the association between PTSS and pain interference (eg, having trouble sleeping or paying attention when in pain).^31^ Pain interference is a measure of pain-related disability and a primary clinical target for individuals with chronic pain. Therefore, the PTSS-driven responses to this experimental pain task may reflect cognitions and behaviors (as occurs with pain interference) that serve to maintain chronic pain conditions in youth.

Mutually maintaining feelings of hyperarousal and threat avoidance correspond to alterations in corticolimbic activity. Continuous activation and deactivation of these corticolimbic regions may lead to structural changes within these brain regions, which have been associated with the chronification of pain.^23,29,30,42,44^ Therefore, by managing PTSS in youth clinically, it may be possible to prevent these corticolimbic changes, which may in turn interrupt the development of chronic pain and comorbid mental health disorders in adolescence and adulthood.

In this study, youth could obtain a high PTSS score on the PTSS scale without having experienced a traumatic event fitting the Diagnostic and Statistical Manual of Mental Disorders criteria. Higher vs lower PTSS scores are relative to the youth included in this study and do not necessarily indicate that youth have met the clinical cutoff for PTSS. A limitation of this study was that concurrent measures of PTSS and sensitization were obtained in youth with chronic pain. Therefore, we could not truly evaluate the influence of trauma symptoms over time on pain sensitization and/or the development and maintenance of chronic pain in youth. Youth were asked about the timing of their most distressing or traumatic event, and 48% of youth identified a traumatic experience that preceded the development of their chronic pain by at least 3 months. However, we do know whether the PTSS related to this event contributed to their chronic pain development. Furthermore, we cannot ascertain from this study whether a different traumatic event from the ones identified preceded and/or contributed to the development of chronic pain in these youth. Moreover, we did not have a comparison group to determine whether our findings were specific to individuals with preexisting chronic pain and trauma. Future research on the relationship and influence of early life trauma on pain and pain symptomology is needed and may lead to the identification and prevention of chronic pain in youth.

The relationships between trauma and pain have been well-established, particularly among adults. However, the mechanisms underlying the relationships between trauma and pain remain unclear, and studies exploring the relationships between trauma symptoms and pain sensitization have revealed conflicting results. While there have been many studies to explore the relationships between trauma and pain sensitization among adults, there appears to be a dearth of knowledge of these relationships in youth. The present study found that greater PTSS was associated with greater state pain catastrophizing both before and after exposure to pain, as well as higher pain thresholds. Posttraumatic stress symptoms appear to be associated with behavioral and physiological responses to acute pain and may underlie the development and maintenance of chronic pain in youth. More research disentangling the longitudinal relationships between trauma symptoms and pain is needed. This research will help to inform early and targeted interventions for high-risk youth, which may prevent the persistence of symptoms into adulthood.

## Disclosures

The authors have no conflicts of interest to declare.

## References

[1] Asmundson GJ Coons MJ Taylor S Katz J. PTSD and the experience of pain: research and clinical implications of shared vulnerability and mutual maintenance models. Can J Psychiatry 2002;47:930–7. 12553128 10.1177/070674370204701004

[2] American Psychiatric Association. Diagnostic and statistical manual of mental disorders, Fifth Edition. Arlington: American Psychiatric Association, 2013.

[3] American Psychiatric Association. Diagnostic and statistical manual of mental disorders: DSM-V. Washington: American Psychiatric Association, 2013.

[4] Birnie KA Noel M Chambers CT von Baeyer CL Fernandez CV. The cold pressor task: is it an ethically acceptable pain research method in children? J Pediatr Psychol 2011;36:1071–81. 20926408 10.1093/jpepsy/jsq092

[5] Birnie KA Petter M Boerner KE Noel M Chambers CT. Contemporary use of the cold pressor task in pediatric pain research: a systematic review of methods. J Pain 2012;13:817–26. 22846592 10.1016/j.jpain.2012.06.005

[6] Bosco MA Gallinati JL Clark ME. Conceptualizing and treating comorbid chronic pain and PTSD. Pain Res Treat 2013:174728. 23819047 10.1155/2013/174728 PMC3684116

[7] Butler RK Finn DP. Stress-induced analgesia. Prog Neurobiol 2009;88:184–202. 19393288 10.1016/j.pneurobio.2009.04.003

[8] Chorpita BF Moffitt CE Gray J. Psychometric properties of the revised child anxiety and depression scale in a clinical sample. Behav Res Ther 2005;43:309–22. 15680928 10.1016/j.brat.2004.02.004

[9] Chorpita BF Yim L Moffitt C Umemoto LA Francis SE. Assessment of symptoms of DSM-IV anxiety and depression in children: a Revised Child Anxiety and Depression Scale. Behav Res Ther 2000;38:835–55. 10937431 10.1016/s0005-7967(99)00130-8

[10] Cooporation I. IBM SPSS statistics for macintosh, version 26.0. Armonk: IBM Corp., 2019.

[11] Defrin R Ginzburg K Solomon Z Polad E Bloch M Govezensky M Schreiber S. Quantitative testing of pain perception in subjects with PTSD--implications for the mechanism of the coexistence between PTSD and chronic pain. PAIN 2008;138:450–9. 18585862 10.1016/j.pain.2008.05.006

[12] Defrin R Schreiber S Ginzburg K. Paradoxical pain perception in posttraumatic stress disorder: the unique role of anxiety and dissociation. J Pain 2015;16:961–70. 26168878 10.1016/j.jpain.2015.06.010

[13] Diatchenko L Slade GD Nackley AG Bhalang K Sigurdsson A Belfer I Goldman D Xu K Shabalina SA Shagin D Max MB Makarov SS Maixner W. Genetic basis for individual variations in pain perception and the development of a chronic pain condition. Hum Mol Genet 2005;14:135–43. 15537663 10.1093/hmg/ddi013

[14] Diener SJ Wessa M Ridder S Lang S Diers M Steil R Flor H. Enhanced stress analgesia to a cognitively demanding task in patients with posttraumatic stress disorder. J Affect Disord 2012;136:1247–51. 21733577 10.1016/j.jad.2011.06.013

[15] Donnelly A Fitzgerald A Shevlin M Dooley B. Investigating the psychometric properties of the Revised Child Anxiety and Depression Scale (RCADS) in a non-clinical sample of Irish adolescents. J Ment Health 2019;28:345–56. 29447056 10.1080/09638237.2018.1437604

[16] Durand H Birnie KA Noel M Vervoort T Goubert L Boerner KE Chambers CT Caes L. State versus trait: validating state assessment of child and parental catastrophic thinking about children's acute pain. J Pain 2017;18:385–95. 27919776 10.1016/j.jpain.2016.11.012

[17] Foa EB Johnson KM Feeny NC Treadwell KR. The Child PTSD Symptom Scale: a preliminary examination of its psychometric properties. J Clin Child Psychol 2001;30:376–84. 11501254 10.1207/S15374424JCCP3003_9

[18] Geuze E Westenberg HG Jochims A de Kloet CS Bohus M Vermetten E Schmahl C. Altered pain processing in veterans with posttraumatic stress disorder. Arch Gen Psychiatry 2007;64:76–85. 17199057 10.1001/archpsyc.64.1.76

[19] Groenewald CB Essner BS Wright D Fesinmeyer MD Palermo TM. The economic costs of chronic pain among a cohort of treatment-seeking adolescents in the United States. J Pain 2014;15:925–33. 24953887 10.1016/j.jpain.2014.06.002 PMC4150826

[20] Harris PA Taylor R Thielke R Payne J Gonzalez N Conde JG. Research electronic data capture (REDCap)—a metadata-driven methodology and workflow process for providing translational research informatics support. J Biomed Inform 2009;42:377–81. 18929686 10.1016/j.jbi.2008.08.010 PMC2700030

[21] Holley AL Wilson AC Noel M Palermo TM. Post-traumatic stress symptoms in children and adolescents with chronic pain: a topical review of the literature and a proposed framework for future research. Eur J Pain 2016;20:1371–83. 27275585 10.1002/ejp.879 PMC5912261

[22] Hotopf M Carr S Mayou R Wadsworth M Wessely S. Why do children have chronic abdominal pain, and what happens to them when they grow up? Population based cohort study. BMJ 1998;316:1196–200. 9552994 10.1136/bmj.316.7139.1196 PMC28520

[23] Hubbard CS Khan SA Xu S Cha M Masri R Seminowicz DA. Behavioral, metabolic and functional brain changes in a rat model of chronic neuropathic pain: a longitudinal MRI study. Neuroimage 2015;107:333–44. 25524649 10.1016/j.neuroimage.2014.12.024

[24] Kasch H Qerama E Bach FW Jensen TS. Reduced cold pressor pain tolerance in non-recovered whiplash patients: a 1-year prospective study. Eur J Pain 2005;9:561–9. 16139185 10.1016/j.ejpain.2004.11.011

[25] King S Chambers CT Huguet A MacNevin RC McGrath PJ Parker L MacDonald AJ. The epidemiology of chronic pain in children and adolescents revisited: a systematic review. PAIN 2011;152:2729–38. 22078064 10.1016/j.pain.2011.07.016

[26] Larsen B Luna B. Adolescence as a neurobiological critical period for the development of higher-order cognition. Neurosci Biobehav Rev 2018;94:179–95. 30201220 10.1016/j.neubiorev.2018.09.005 PMC6526538

[27] Ludascher P Valerius G Stiglmayr C Mauchnik J Lanius RA Bohus M Schmahl C. Pain sensitivity and neural processing during dissociative states in patients with borderline personality disorder with and without comorbid posttraumatic stress disorder: a pilot study. J Psychiatry Neurosci 2010;35:177–84. 20420768 10.1503/jpn.090022 PMC2861134

[28] Manning BH. A lateralized deficit in morphine antinociception after unilateral inactivation of the central amygdala. J Neurosci 1998;18:9453–70. 9801383 10.1523/JNEUROSCI.18-22-09453.1998 PMC6792902

[29] Miller JV Andre Q Timmers I Simons L Rasic N Lebel C Noel M. Subclinical post-traumatic stress symptomology and brain structure in youth with chronic headaches. Neuroimage Clin 2021;30:102627. 33812302 10.1016/j.nicl.2021.102627 PMC8053811

[30] Mutso AA Petre B Huang L Baliki MN Torbey S Herrmann KM Schnitzer TJ Apkarian AV. Reorganization of hippocampal functional connectivity with transition to chronic back pain. J Neurophysiol 2014;111:1065–76. 24335219 10.1152/jn.00611.2013 PMC3949236

[31] Neville A Soltani S Pavlova M Noel M. Unravelling the relationship between parent and child PTSD and pediatric chronic pain: the mediating role of pain catastrophizing. J Pain 2018;19:196–206. 29081370 10.1016/j.jpain.2017.10.004

[32] Nishith P Griffin MG Poth TL. Stress-induced analgesia: prediction of posttraumatic stress symptoms in battered versus nonbattered women. Biol Psychiatry 2002;51:867–74. 12022959 10.1016/s0006-3223(01)01346-4

[33] Noel M Groenewald CB Beals-Erickson SE Gebert JT Palermo TM. Chronic pain in adolescence and internalizing mental health disorders: a nationally representative study. PAIN 2016;157:1333–8. 26901806 10.1097/j.pain.0000000000000522 PMC4939835

[34] Noel M Wilson AC Holley AL Durkin L Patton M Palermo TM. Posttraumatic stress disorder symptoms in youth with vs without chronic pain. PAIN 2016;157:2277–84. 27276275 10.1097/j.pain.0000000000000642 PMC5028262

[35] Otis JD Keane TM Kerns RD. An examination of the relationship between chronic pain and post-traumatic stress disorder. J Rehabil Res Dev 2003;40:397–405. 15080224 10.1682/jrrd.2003.09.0397

[36] Palermo TM. Impact of recurrent and chronic pain on child and family daily functioning: a critical review of the literature. J Dev Behav Pediatr 2000;21:58–69. 10706352 10.1097/00004703-200002000-00011

[37] Palermo TM Eccleston C. Parents of children and adolescents with chronic pain. PAIN 2009;146:15–17. 19482426 10.1016/j.pain.2009.05.009 PMC2760649

[38] Petrovic P Carlsson K Petersson KM Hansson P Ingvar M. Context-dependent deactivation of the amygdala during pain. J Cogn Neurosci 2004;16:1289–301. 15453980 10.1162/0898929041920469

[39] Powers A Fani N Pallos A Stevens J Ressler KJ Bradley B. Childhood abuse and the experience of pain in adulthood: the mediating effects of PTSD and emotion dysregulation on pain levels and pain-related functional impairment. Psychosomatics 2014;55:491–9. 24360527 10.1016/j.psym.2013.10.004 PMC3997632

[40] Raphael KG Widom CS. Post-traumatic stress disorder moderates the relation between documented childhood victimization and pain 30 years later. PAIN 2011;152:163–9. 21050659 10.1016/j.pain.2010.10.014 PMC3053034

[41] Shelby GD Shirkey KC Sherman AL Beck JE Haman K Shears AR Horst SN Smith CA Garber J Walker LS. Functional abdominal pain in childhood and long-term vulnerability to anxiety disorders. Pediatrics 2013;132:475–82. 23940244 10.1542/peds.2012-2191 PMC3876748

[42] Simons LE Pielech M Erpelding N Linnman C Moulton E Sava S Lebel A Serrano P Sethna N Berde C Becerra L Borsook D. The responsive amygdala: treatment-induced alterations in functional connectivity in pediatric complex regional pain syndrome. PAIN 2014;155:1727–42. 24861582 10.1016/j.pain.2014.05.023 PMC4157948

[43] Tesarz J Baumeister D Andersen TE Vaegter HB. Pain perception and processing in individuals with posttraumatic stress disorder: a systematic review with meta-analysis. Pain Rep 2020;5:e849. 33490843 10.1097/PR9.0000000000000849 PMC7808684

[44] Vachon-Presseau E Tetreault P Petre B Huang L Berger SE Torbey S Baria AT Mansour AR Hashmi JA Griffith JW Comasco E Schnitzer TJ Baliki MN Apkarian AV. Corticolimbic anatomical characteristics predetermine risk for chronic pain. Brain 2016;139:1958–70. 27190016 10.1093/brain/aww100 PMC4939699

[45] van der Kolk BA Greenberg MS Orr SP Pitman RK. Endogenous opioids, stress induced analgesia, and posttraumatic stress disorder. Psychopharmacol Bull 1989;25:417–21. 2626517

[46] Vinall J Pavlova M Asmundson GJ Rasic N Noel M. Mental health comorbidities in pediatric chronic pain: a narrative review of epidemiology, models, neurobiological mechanisms and treatment. Children (Basel) 2016;3:40. 10.3390/children3040040 PMC5184815 27918444

[47] von Baeyer CL Piira T Chambers CT Trapanotto M Zeltzer LK. Guidelines for the cold pressor task as an experimental pain stimulus for use with children. J Pain 2005;6:218–27. 15820909 10.1016/j.jpain.2005.01.349

[48] Walker LS Sherman AL Bruehl S Garber J Smith CA. Functional abdominal pain patient subtypes in childhood predict functional gastrointestinal disorders with chronic pain and psychiatric comorbidities in adolescence and adulthood. PAIN 2012;153:1798–806. 22721910 10.1016/j.pain.2012.03.026 PMC3413740

